# Recurrent Gastrointestinal Pseudo-obstruction Because of Well-Differentiated Duodenal Neuroendocrine Tumor

**DOI:** 10.14309/crj.0000000000000910

**Published:** 2022-12-26

**Authors:** Ruchi Sharma, Hammad Zafar, Scott K. Sherman, Fadi Niyazi

**Affiliations:** 1 Department of Internal Medicine, University of Iowa Hospital and Carver College of Medicine, Iowa City, IA; 2 Department of Surgical Oncology and Endocrine Surgery, University of Iowa Hospital and Carver College of Medicine, Iowa City, IA; 3 Department of Gastroenterology and Hepatology, University of Iowa Hospital and Carver College of Medicine, Iowa City, IA

## Abstract

A 56-year-old man presented with recurrent gastrointestinal obstruction. Computed tomography showed fluid-filled, distended stomach, small intestine, and large intestine. Extensive workup including esophagogastroduodenoscopy, colonoscopy, magnetic resonance enterography, push enteroscopy, and video capsule enteroscopy showed no mechanical obstruction. Endoscopic ultrasound–guided biopsy of peripancreatic nodes detected on ^18^F-fluorodeoxyglucose positron emission tomography revealed a duodenal neuroendocrine tumor. The lesion showed intense uptake on gallium-68 DOTATOC positron emission tomography-computed tomography scan. The patient underwent surgical resection of the tumor with resolution of bowel obstruction events. He had elevated pancreatic polypeptide levels, which are known to delay gastric emptying and could explain his symptoms.

## INTRODUCTION

Neuroendocrine tumors (NET) are a rare cause of bowel obstruction. At the time of diagnosis, most tumors have metastasized. Knowledge of unique diagnostic methods is imperative to timely diagnosis and management.

## CASE REPORT

A 56-year-old man was transferred to our hospital after over 10 admissions to an outside facility for gastrointestinal (GI) obstruction over the preceding year. He had lost 50 pounds of weight during this period. He had no history of abdominal surgeries. He presented with epigastric abdominal pain, distension, nausea, and emesis. He was managed nonoperatively with nasogastric tube placement and bowel rest.

**Figure 1.**
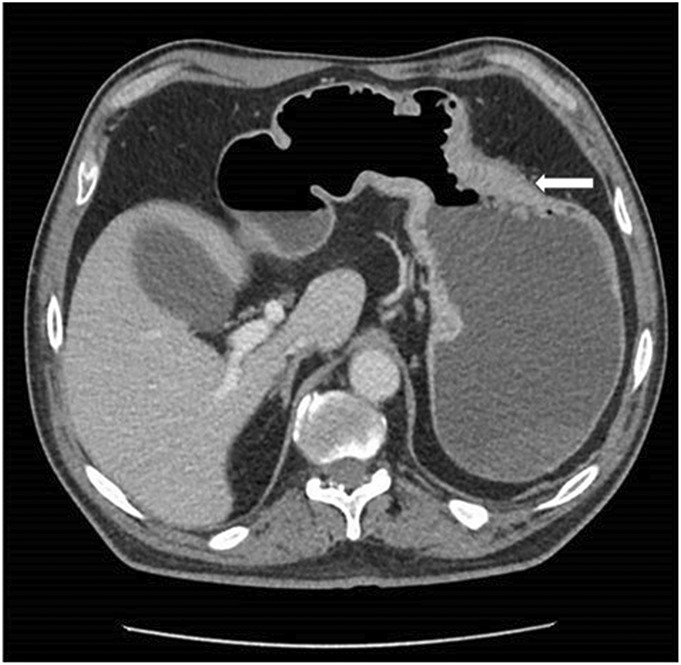
Computed tomography of the abdomen with dilated stomach and duodenum with decompressed distal small intestine (arrow).

Previous computed tomography (CT) scans of the abdomen showed variable distension of the stomach, or fluid-filled small intestine with concern for transition point at the ileum, or distended large intestine. He had formerly undergone 2 esophagogastroduodenoscopies, which did not reveal gastric outlet obstruction. His current CT abdomen showed a fluid-filled stomach and duodenum, suggestive of gastric outlet obstruction (Figure 1). Magnetic resonance enterography (MRE) did not show obstruction, but demonstrated a 2.1 × 2.3-cm right mesenteric nodule with similar enhancement as the spleen, raising the possibility of ectopic splenic tissue, and a 0.7-cm nodule posterior to the uncinate process.

Push enteroscopy revealed esophagitis and severe aphthous ulcerations in the small intestine, but no obstruction. Colonoscopy did not reveal any obstructive pathology. Video capsule enterography revealed erythematous mucosa with villous blunting and nonobstructive luminal narrowing in the duodenum through which the capsule passed easily.

An ^18^F-fluorodeoxyglucose positron emission tomography (^18^FDG PET)/CT scan was performed to further evaluate the mesenteric lesion seen on MRE. It showed multiple mild to moderately FDG avid cervical lymph nodes (LN), the largest a 4-cm left supraclavicular LN. It redemonstrated the soft-tissue lesion anterior to the duodenum with mild to moderate FDG uptake and multiple borderline retroperitoneal LN. There was no abnormal uptake in the intestines, which could explain the recurrent obstruction. Ultrasound-guided biopsy of the left supraclavicular LN revealed a hemangioma.

Endoscopic ultrasound performed to investigate the FDG avid mass showed a hypoechoic lesion, 18 mm in maximum diameter at the porta hepatis correlating with the suspicious findings on the ^18^FDG PET/CT scan. The visualized liver was normal.

**Figure 2.**
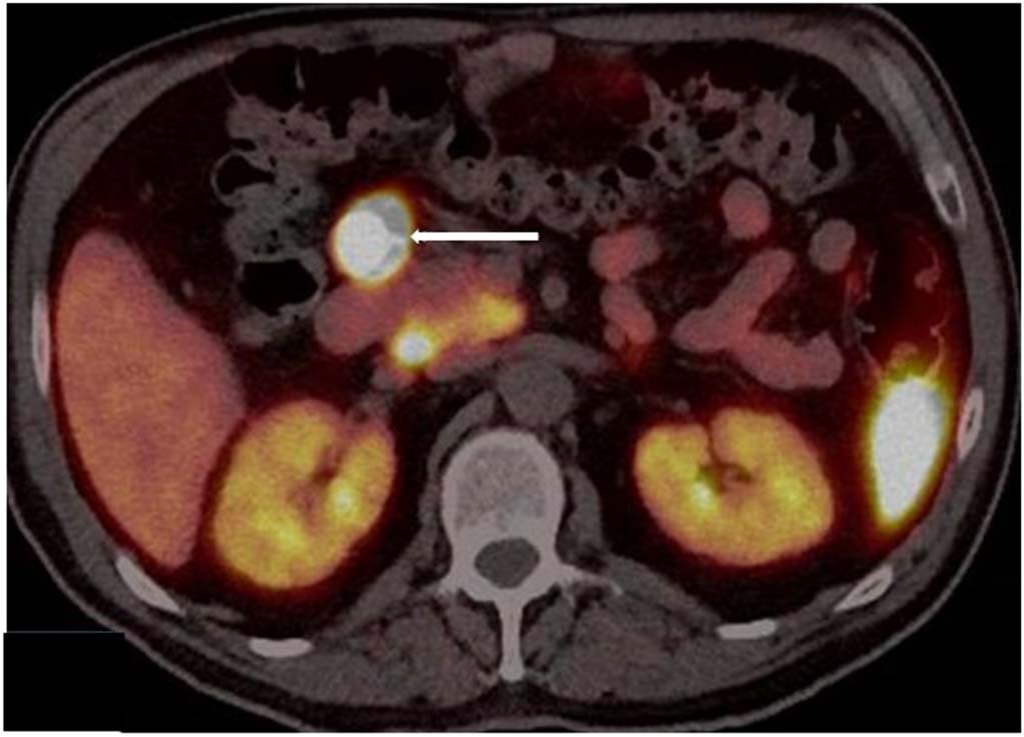
Ga-68 DOTATOC positron emission tomography/computed tomography scan showing somatostatin receptor–positive peripancreatic node (arrow).

Fine-needle biopsy of the porta hepatis lesion revealed a well-differentiated NET. gallium-68 DOTATOC PET/CT scan showed a 2.3 × 2.2-cm intensely somatostatin receptor positive lesion anterior to the duodenum and 2 intensely positive peripancreatic nodules adjacent to the uncinate process, larger measuring 1.4 cm (Figure 2). Plasma concentrations of NET-related hormones included pancreatic polypeptide (PP) 2,145 pg/mL (0–435), chromogranin A 1,458 ng/mL (0–103), serotonin 278 ng/mL (50–200), gastrin 208 ng/mL (0–100), pancreastatin 88 pg/mL (10–135), and neurokinin A <5 pg/mL (<40).

On exploratory laparotomy, he was found to have a subcentimeter proximal duodenal tumor with central puckering; 2 large LN, 1 anterior, and 1 posterior to the second part of the duodenum; and a distended gallbladder with gallstones. No liver mass was discerned on palpation or intraoperative ultrasound. He underwent a partial duodenal resection with primary repair, resection of peripancreatic LN, and cholecystectomy. Surgical pathology showed a 1-cm full-thickness, nonobstructive well-differentiated, grade 1 (Ki-67 2.4%) duodenal NET with metastatic peripancreatic LN; stage pT1N1. The postoperative course was uneventful. Because there was no residual tumor, he was not started on octreotide. Repeat gallium-68 PET DOTATOC scan was negative for residual disease. Postoperatively, serotonin and chromogranin A levels normalized (197 ng/mL and 89 ng/mL, respectively), whereas PP decreased to 1,510 pg/mL. Since discharge, the patient has not developed any further episodes of mechanical small bowel obstruction and has gained 33 pounds of weight.

## DISCUSSION

NET arise from neuroendocrine cells and are most commonly found in the lung, small intestine, and pancreas.^1^ Because of improved diagnostic modalities, their incidence has increased over time. They are, however, rare, and knowledge of appropriate diagnostic modalities is of utmost importance for timely diagnosis and treatment because they can often be missed on conventional imaging.^2^ At the time of diagnosis, most patients have metastatic disease.^3^

We could not pinpoint the exact etiology of recurrent obstructive events in this patient. NET presenting with bowel obstruction usually result from a mechanical cause.^4,5^ In the present case, despite convincing symptomatology and CT findings with intestinal dilation and a transition point, extensive workup including esophagogastroduodenoscopies, colonoscopy, MRE, push enteroscopy, and video capsule enterography failed to show obstructive pathology. No obstruction was noted on surgical exploration.

Levels of PP and chromogranin A were markedly elevated. PP can delay gastric emptying, although this effect has been questioned.^6,7^ Chromogranin A has no effect on intestinal motility.^8^ Somatostatin can reduce GI motility, but levels were normal.^9^ Serotonin and gastrin levels were mildly elevated. The patient had evidence of gastritis/esophagitis. However, gastrin levels were not high enough to suggest Zollinger-Ellison syndrome. Moreover, the patient was on a proton pump inhibitor.

Metastatic LN in NET can cause mesenteric fibrosis through the release of angiogenic and fibroblast-inducing mediators.^10^ Hence, these tumors can be associated with a mesenteric desmoplastic reaction, resulting in GI obstruction.^3,11^ A mesenteric inflammatory reaction that, if allowed to persist, might have progressed to a desmoplasia could also cause recurrent obstructions in this patient. However, no fibrosis was appreciated on exploratory laparotomy. We believe the NET were the cause of bowel obstruction in this patient because his symptoms improved after resection and he regained weight.

Most duodenal NET <2 cm do not have nodal metastases and may be resected endoscopically.^12^ In this case, known nodal metastases precluded endoscopic resection. Moreover, the primary tumor was not definitively identified until laparotomy. Although a Whipple procedure represents the standard therapy and would provide a more complete lymphadenectomy, the patient's nutritional status placed him at high risk of complications from such an extensive operation. The tumor's small size and antimesenteric location made a lower-risk local resection feasible without compromising later radical resection if needed after improvement in nutrition.

Clinical persistence, appropriate diagnostics, and attention to detail were instrumental in his diagnosis and treatment. Because NET can often lead to bowel obstruction because of neurohormonal effects or desmoplastic reaction, and they are not always detectable on conventional imaging modalities such as magnetic resonance imaging or CT scans, a gallium-68 PET DOTATOC scan may be a reasonable investigation for a patient presenting with recurrent bowel obstruction and no identifiable cause on routine imaging.

## DISCLOSURES

Author contributions: R. Sharma: conception, analysis and interpretation of data, and writing manuscript, accountable for all aspects of work. H. Zafar: analysis and interpretation of data. SK Sherman: critically revising manuscript. F. Niyazi: conception, critical review, accountable for all aspects of work, and is the article guarantor.

Financial disclosure: None to report.

Informed consent was obtained for this case report.
